# Oxytocin Receptor Binding Sites in the Periphery of the Neonatal Prairie Vole

**DOI:** 10.3389/fnins.2019.00474

**Published:** 2019-05-24

**Authors:** Maria A. Greenwood, Elizabeth A. D. Hammock

**Affiliations:** ^1^Program in Neuroscience, Florida State University, Tallahassee, FL, United States; ^2^Department of Psychology, Florida State University, Tallahassee, FL, United States

**Keywords:** oxytocin receptor (OXTR), prairie vole (*Microtus ochrogaster*), neonate, autoradiography, oxytocin

## Abstract

The oxytocin receptor (OXTR) has been observed in the periphery of neonatal C57BL/6J mice (*Mus musculus*), including facial regions and the anogenital area. In those studies, ligand specificity was confirmed with a congenital OXTR knockout mouse as well as competitive binding techniques. The aim of this study was to determine if OXTR is present in the same peripheral sites in the neonatal prairie vole (*Microtus ochrogaster*) for cross-species comparisons. Receptor autoradiography was performed on 20 μm sagittal sections of whole postnatal day 0 (P0) male and female prairie voles using the ^125^iodinated-ornithine vasotocin ([^125^I]-OVTA) radioligand. A competition binding assay was used to assess the selectivity of [^125^I]-OVTA for peripheral OXTR. Radioactive ligand (0.05 nM [^125^I]-OVTA) was competed against concentrations of 0 and 1000 nM excess unlabeled oxytocin (OXT). Previously identified regions of significant OXTR ligand binding in the mouse were analyzed for comparison: rostral and lateral periodontium, olfactory epithelium, ciliary bodies of the eye, whisker pads, adrenal gland, and anogenital area. We also evaluated the liver and scapular brown adipose tissue, which displayed strong but non-specific signal on film in mice. While there were some areas that showed conserved OXTR ligand binding in the prairie vole (e.g., ciliary body of the eye and the anogenital area), areas showing OXTR ligand binding in the neonatal prairie vole were not identical to OXTR ligand binding in the periphery of the C57BL/6J neonatal mouse. Further, some of the regions measured in the prairie vole suggest sex differences in OXTR ligand binding. Collectively, as is well-established in the central nervous system, these data indicate that patterns of OXTR ligand binding in the infant periphery are species-specific.

## Introduction

Oxytocin (OXT) has a well-established role as a neuropeptide known to regulate maternal behavior via central pathways as well as peripheral physiology relating to parturition, specifically the milk let-down reflex and uterine muscle contractions. OXT receptors (OXTR) are found in the brain and in numerous mammalian peripheral tissues in males and females (Gimpl and Fahrenholz, 2001), and the activation of these OXTR have been shown to facilitate the transition to parenthood (Pedersen and Prange, 1979, 1985; Russell et al., 2001; Marlin et al., 2015). While research has primarily focused on the role of OXT to modulate parental behaviors in adults, less is known about how OXT may shape infant development.

Knowing where OXT may act in the neonate is an important part of understanding its role in shaping infant development. Previous work from our lab examined OXTR in the periphery of neonatal C57Bl/6J mice, finding OXTR ligand binding was regionally specific in wild-type mice and absent in the *Oxtr* knockout mouse (Greenwood and Hammock, 2017). The goal of the current study was to assess regions previously identified in the mouse for possible OXTR ligand binding in the neonatal prairie vole (*Microtus ochrogaster*). Young prairie voles have demonstrated a sensitivity to the administration of exogenous OXT during the perinatal period (Cushing et al., 2003; Carter et al., 2009; Miller and Caldwell, 2015), including lasting changes to cytoarchitecture of the developing brain (Bales et al., 2007; Seelke et al., 2016). This, in conjunction with the breadth of OXT-focused research in adult prairie voles, suggests prairie voles are a good model organism for the investigation of peripheral OXTR. Research to date suggests significant differences in OXTR patterns in the brain across different species during development (Vaidyanathan and Hammock, 2016) and in adulthood (Young, 1999), including established differences between the laboratory mouse (*Mus musculus*) (Hammock and Levitt, 2013) and the prairie vole (Duchemin et al., 2017). Data within this paper will expand upon these species comparisons by investigating OXTR ligand binding in the periphery of the neonatal prairie vole. Individual regions of interest were chosen based on our prior report of the distribution of OXTR ligand binding in neonatal mice. Therefore, the purpose of the current study was to determine if regions found to demonstrate OXTR ligand binding in the neonatal mouse also had detectible levels of OXTR in prairie vole neonates.

## Materials and Methods

### Subjects

Prairie voles (*Microtus ochrogaster*) were generously donated by Dr. Zuoxin Wang (Department of Psychology, Program in Neuroscience, Florida State University). All procedures were performed under protocols approved by the Institutional Animal Care and Use Committee of Florida State University in accordance with state and federal guidelines (Guide for the Care and Use of Laboratory Animals of the National Institutes of Health). Pregnancies were not timed; breeding pairs were checked daily, with the first appearance of a litter established as postnatal day 0 (P0). On P0, parents were removed from the homecage, litters were euthanized under prolonged CO_2_ exposure, and whole-body tissue was frozen in dry ice. Samples were collected from three separate litters, one female and one male were taken from each litter for this study. Male and female pups were collected, as identified by visual inspection on P0. Sex was verified by anatomical inspection of genitalia on slides after sectioning. Specimens were stored at -80°C until cryosectioning.

### Receptor Autoradiography

Tissue was cryosectioned in 8 series at 20 μm in the sagittal plane and mounted on SuperFrost Plus microscope slides (Fisher Scientific). Sections were stored at -80°C until receptor autoradiography was performed. Receptor autoradiography followed standard methods (Hammock and Levitt, 2013) using 0.05 nM ^125^I labeled OXTR ligand (iodinated-ornithine vasotocin analog NEX254 [OVTA]; (Elands et al., 1988a,b); [^125^I]-OVTA, Perkin-Elmer, Waltham, MA, United States). A competitive binding autoradiography protocol was used to assess non-specific ligand binding in the periphery of the neonatal prairie vole. For this competition assay, unlabeled OXT peptide (oxytocin acetate salt hydrate, cat# O6379, Sigma-Aldrich, St. Louis, MO, United States) was added to the radioactive tracer at a concentration of 1000 nM on a separate series of adjacent sections. The concentration of unlabeled OXT peptide competed with ^125^I-OVTA for receptors, to decrease the visible signal in regions containing OXTR. This concentration was chosen based on our prior work in mice (Greenwood and Hammock, 2017) demonstrating that 1000 nM excess unlabeled OXT was sufficient to occupy all the available receptors in the mouse periphery. Autoradiographic films (Kodak Biomax MR film, Carestream Health, Inc., Rochester, NY, United States) were exposed to slides and ^14^C autoradiographic standards (ARC-0146; American Radiolabeled Chemicals, St. Louis, MO, United States) for approximately 72 h before developing (Mini-Medical/90 X-ray film processor, AFP Imaging, Mount Kisco, NY, United States).

**Table 1:** Signal significantly (*p* < 0.05) higher than zero was observed in the olfactory epithelium, ciliary bodies of the eye, and the anogenital region.

Region of Interest	One-sample *t*-test for region of interest		
	
	Mean μCi/g ( ± SEM)	*t* (df)	Significant?	Cohen’s *d*
Rostral Tooth	0.0116 ± 0.0055	2.110 (5)	No (*p* = 0.0887)	0.8614
Whisker Pads	0.0027 ± 0.0122	0.222 (5)	No (*p* = 0.8329)	0.0907
Lateral Tooth	0.0143 ± 0.0090	1.586 (5)	No (*p* = 0.1736)	0.6476
Olfactory Epithelium	0.0125 ± 0.0041	3.051 (5)	Yes (*p* = 0.0284)	1.2457
Ciliary Bodies	0.0922 ± 0.0091	10.090 (5)	Yes (*p* = 0.0002)	4.1214
Adrenal Gland	0.0646 ± 0.0330	1.956 (5)	No (*p* = 0.1078)	0.7987
Anogenital	0.0296 ± 0.0043	6.786 (5)	Yes (*p* = 0.0011)	2.7715
Liver	0.0166 ± 0.0115	1.441 (5)	No (*p* = 0.2092)	0.5883
Brown Adipose Tissue	0.0919 ± 0.0434	2.114 (5)	No (*p* = 0.0882)	0.8631


*Estimated effect size was calculated using Cohen’s *d*.*

**Table 2:** Significant differences (*p* < 0.05) between sexes were observed in the anogenital area and the brown adipose tissue.

Region of Interest	Two-sample *t*-test for sex differences				
	
	Male Mean ( ± SEM)	Female Mean ( ± SEM)	*t* (*df*)	Significant?	Cohen’s *d*
Rostral Tooth	0.0153 ± 0.0106	0.0079 ± 0.0049	0.6207 (4)	No (*p* = 0.5684)	0.5071
Whisker Pads	-0.0074 ± 0.0111	0.0129 ± 0.0228	0.7999 (4)	No (*p* = 0.4686)	0.6531
Lateral Tooth	0.0189 ± 0.0158	0.0097 ± 0.0116	0.4687 (4)	No (*p* = 0.6637)	0.3827
Olfactory Epithelium	0.0086 ± 0.0074	0.0164 ± 0.0037	0.9442 (4)	No (*p* = 0.3985)	0.7707
Ciliary Bodies	0.0936 ± 0.0154	0.0909 ± 0.0133	0.1324 (4)	No (*p* = 0.9011)	0.1080
Adrenal Gland	0.0209 ± 0.0402	0.1083 ± 0.0439	1.469 (4)	No (*p* = 0.2159)	1.1991
Anogenital	0.0213 ± 0.0039	0.0379 ± 0.0037	3.194 (4)	Yes (*p* = 0.0331)	2.6091
Liver	0.0172 ± 0.0229	0.0161 ± 0.0118	0.0427 (4)	No (*p* = 0.9680)	0.0348
Brown Adipose Tissue	0.0098 ± 0.0328	0.1741 ± 0.0404	3.16(4)	Yes (*p* = 0.0342)	2.5801


*Estimated effect size was calculated using Cohen’s *d*.*

### Image Analysis

After autoradiography, all slides were post-processed with cresyl violet stain for unbiased region of interest measurements. Briefly, slides were incubated in 0.5% cresyl violet solution at 37°C for 5 min, rinsed in distilled water, differentiated in 95% ethanol alcohol in two 5-minute washes, and dehydrated in 100% ethanol alcohol in two 5-minute washes. Finally, slides were cleared in CitriSolv (Decon Labs Inc., King of Prussia, PA, United States) in two 5-minute washes and cover-slipped. Films and stained slides were scanned on a flatbed scanner at 1200 dpi (EPSON, Epson Perfection V600 Photo). Regions of interest were identified on post-processed slides and then measurements were collected from corresponding film images. Quantifications were recorded in ImageJ (NIH, Bethesda, MD, United States) using the brush-selection tool, from three consecutive sections within each animal. Local background values were obtained from non-tissue background of the slide adjacent to the region of interest and measured in a 20 × 20 pixel region, then subtracted from the region of interest values to generate local densitometry values. For quantification, no image adjustments were made with the exception of image inversion so that higher numbers represented more dense binding. Ligand binding values in μCi/gram were then calculated by interpolation “interp1,” MatLab 9.0.0 (TheMathworks, Natick, MA, United States) to the linear range of the ^14^C autoradiographic standard on the same film (Miller and Zahniser, 1987).

To determine the amount of signal on film attributable to radioactive ligand binding at OXTR, we calculated a net signal as the difference between competed and un-competed adjacent sections:

net OXTR signal = (binding density with no competition) – (binding density with 1000 nM OXT competition).

Composite images were created using the TurboReg (Thevenez et al., 1998) plugin for ImageJ using the rigid-body alignment algorithm. For pseudocolor composites, the autoradiography images were adjusted for contrast and brightness to minimize the appearance of the film background.

### Statistical Analysis

This study sought to determine if the net difference between our 0 nM autoradiograms and the 1000 nM autoradiograms was significantly different from zero as our *a priori* threshold for determining specific OXTR ligand binding. Regions were analyzed to determine if they had specific OXTR binding (different from zero) by a two-tailed one-sample *t*-test of net signal values, with sexes combined (*n* = 6) for each region of interest analyzed. Our prior work in mice yielded effect sizes greater than or equal to Cohen’s *d* of 1.7. Power analysis of the same sample size for this study indicates 80% power at the alpha 0.05 level to detect effect sizes greater than or equal to 1.2 (Cohen’s *d*). A stringent two-tailed test was chosen because for some samples and regions the net signal was less than zero. Estimated effect size was calculated using Cohen’s *d*, [*d* = mean difference / standard deviation]. The possibility of robust sex differences was subsequently explored using a two-sample Student’s *t*-test, Males (*n* = 3) and Females (*n* = 3), for each region of interest. Estimated effect size for sex differences using Cohen’s *d*, (*d* = Mean difference / pooled standard deviation); Pooled standard deviation for equal group sizes = √(SD_1_^2^ + SD_2_^2^)/2.

## Results

First, we evaluated ligand binding across males and females combined to determine if neonatal voles showed evidence of ligand binding in the regions of interest based on our prior work in C57Bl/6J mice. Next, we determined if there was suggestive evidence of robust sex differences in the net ligand binding values. Raw data are available as Supplementary Material.

**FIGURE 1:**
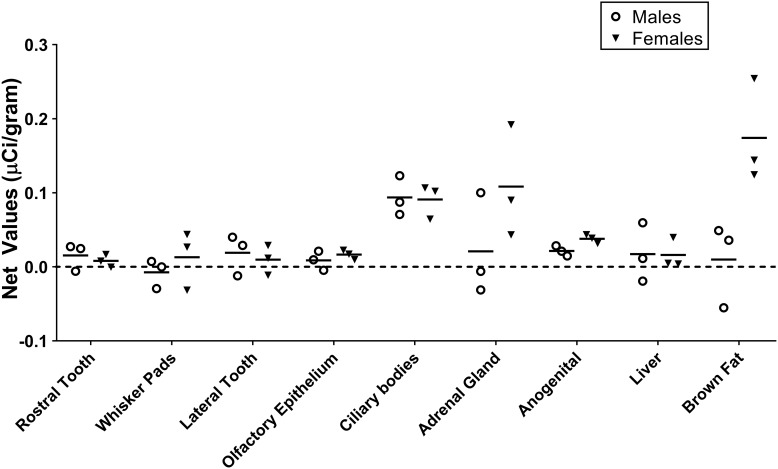
Quantification of receptor autoradiography for net μCi/gram OXTR in the peripheral tissue of neonatal *Microtus ochrogaster.* The net OXTR binding values (net = density of 0–1000 nM) for all regions in prairie voles with specific OXTR ligand binding are compared against a common *y*-axis. Group means and individual subject data are plotted males (open circles), females (filled triangles).

**FIGURE 2:**
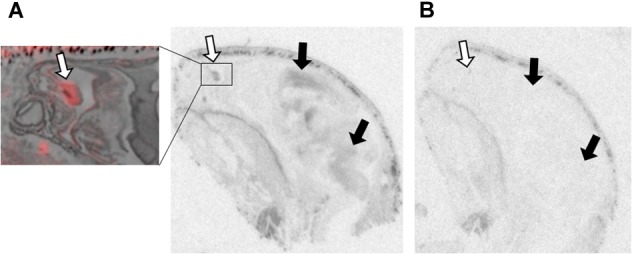
OXTR ligand binding in the olfactory epithelium of neonatal prairie voles. Pseudo-color composites (OXTR in red, cresyl violet counterstain in gray) of neonatal prairie vole with 0 nM OXT competition. White arrows indicate nasal turbinates; quantifications were measured from surface area of nasal epithelium. Black arrows indicate brain regions with **(A)** specific OVTA-ligand binding and **(B)** an adjacent slice with 1000 nM unlabeled OXT competition.

**FIGURE 3:**
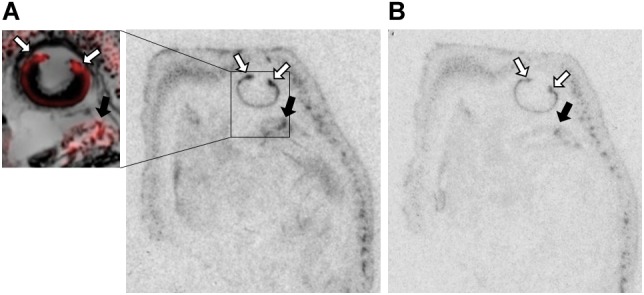
OXTR ligand binding in the ciliary bodies of the eye of neonatal prairie voles. **(A)** Pseudo-color composites (OXTR in red, cresyl violet counterstain in gray) of neonatal prairie vole with 0 nM OXT competition. White arrows indicate ciliary bodies; quantifications were measured at the ciliary bodies only; no measures were taken from the posterior retina. Black arrows indicate brain tissue. **(B)** 1000 nM unlabeled OXT competes off ligand binding in both the ciliary body and the brain.

**FIGURE 4:**
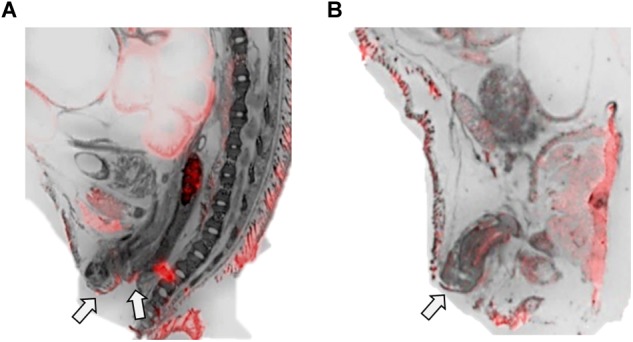
OXTR ligand binding in the anogenital area of neonatal prairie voles. Pseudo-color composites (OXTR in red, cresyl violet counterstain in gray) of **(A)** female neonatal prairie vole with 0 nM OXT competition and **(B)** male neonatal prairie vole with 0 nM OXT competition White arrows indicate areas where quantifications were measured. Females did not display robust OXTR signal visible to the naked eye in the vaginal canal, only on external anogenital tissue. Males, however, displayed OXTR signal on internal and external genital tissue. Quantifications for this study only measured external anogenital tissue.

**FIGURE 5:**
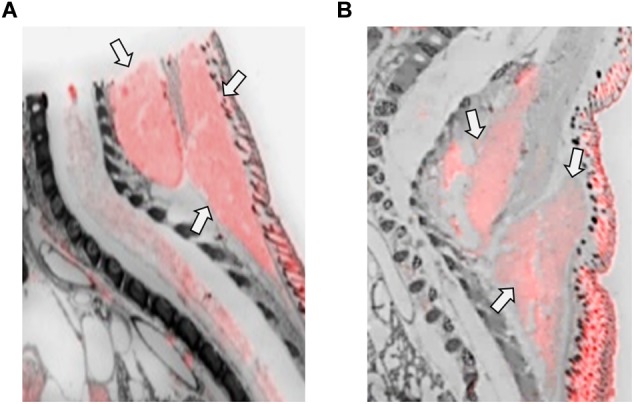
OXTR ligand binding in the brown adipose tissue of neonatal prairie voles. Pseudo-color composites (OXTR in red, cresyl violet counterstain in gray) of **(A)** female neonatal prairie vole with 0 nM OXT competition and **(B)** male neonatal prairie vole with 0 nM OXT competition. Females displayed significantly higher OXTR net signal than males.

Table 1 summarizes the statistical results to confirm the presence of robust OXTR in specific peripheral sites in the prairie vole neonate. Table 2 summarizes the exploratory analysis for potential large sex differences in OXTR ligand binding in the prairie vole neonatal periphery. Figure 1 is a graphical representation of all statistics summarized in Tables 1, 2. A one sample *t*-test analyzing the difference from zero in net signal revealed a significant difference from zero in the olfactory epithelium (Figure 2) [*t* = 3.051, *df* = 5, *p* = 0.0284] with no significant difference between sexes [*t* = 0.9442, *df* = 4, *p* = 0.3985], a significant difference from zero in the ciliary bodies of the eye (Figure 3) [*t* = 10.090, *df* = 5, *p* = 0.0002] with no significant difference between sexes [*t* = 0.1324, *df* = 4, *p* = 0.9011], a significant difference from zero in the anogenital area (Figure 4) [*t* = 6.786, *df* = 5, *p* = 0.0011] with a significant difference between males and females [*t* = 3.194, *df* = 4, *p* = 0.0331], no significant difference from zero in the brown adipose tissue (Figure 5) [*t* = 2.114, *df* = 5, *p* = 0.0882] however, a significant difference between males and females [*t* = 3.16, *df* = 4, *p* = 0.00342].

## Discussion

Data in this paper provide evidence of OXTR ligand binding in the periphery of neonatal prairie voles, with a limited focus on areas previously investigated in mice (Greenwood and Hammock, 2017). These results provide evidence for potential peripheral targets for OXT, as well as important target regions for research on the role of OXT in neonatal development. The use of competition binding techniques provided valuable information regarding the specificity of the radioligand in previously unconfirmed tissues that produce intense signal but do not appear to reflect robust OXTR. Within the regions assessed, our results suggest the presence of specific OXTR in the ciliary bodies of the eye, the olfactory epithelium, and the anogenital area of both male and female prairie vole neonates, with more OXTR in the anogenital area of female neonates. Finally, neonatal female but not male prairie voles displayed evidence of OXTR in scapular brown adipose tissue.

The overall ligand binding of OXTR in the periphery of the neonatal vole was lower across all regions in this analysis when compared to previous finding the periphery of the neonatal C57Bl/6J mouse (Greenwood and Hammock, 2017). However, there is sufficient literature supporting the specificity of [^125^I]-OVTA for OXTRs in both species in the brain (Elands et al., 1988b; Insel and Shapiro, 1992; Hammock and Levitt, 2013). For example, brain ligand binding in all subjects was completely abolished with 1000 nM competition (qualitative evidence in Figure 2). Significant differences have been reported between species and between sexes within a species in centrally expressed OXTRs, reflecting differences in behaviors regulated by OXT (Insel et al., 1991; Young, 1999; Dumais and Veenema, 2016; Freeman and Young, 2016). Regions selected for measurement in this study were not designed to be an exhaustive list, but to provide comparative data for regions measured in the mouse. This does not preclude the presence of OXTRs in other peripheral tissues, but it does demonstrate species differences in this comparison furthered detailed in this section.

In contrast to OXTR ligand binding observed previously in mice, prairie voles displayed non-significant signal in the region of the rostral periodontium, or lower incisor beneath the gum tissue. Like other species within the *Rodentia* order, both mice and prairie voles produce continuously growing incisors (Jheon et al., 2015). However, a main difference between the two species is the early emergence of “milk teeth” in prairie voles (Stallings, 1990), which are not found in mice. Previous findings support the role of OXT as an integral anabolic hormone acting specifically in the periphery, not centrally (Tamma et al., 2009), for bone development in utero (Liu et al., 2009) as well as *in vitro* evidence for skeletal maintenance throughout the lifetime (Elabd et al., 2008; Tamma et al., 2009). A role for OXT in the skeletal development of prairie voles has been unexplored, and data within this paper shows no significant OXTR ligand binding on or adjacent to layers of dentition. However, the high variability of the data within this region suggest that with a larger sample size, a low quantity of OXTR in this area could be detected. Similarly, the lateral periodontium, tissue that is expected to be on or adjacent to molar teeth, showed no significant signal in prairie voles. This is in contrast to significant OXTR signal present in the mouse (Greenwood and Hammock, 2017). Differential ligand binding of OXTR in this region between species may suggest species differences in the regulatory role of OXT signaling in these species, and perhaps more precisely different sensitive periods for the role of OXT as a developmental regulator. Further research is warranted on the role of OXT signaling in tooth development and possibly mediating oral nursing behaviors in the infant.

Whisker pads were quantified by measuring the site of individual vibrissae on the whisker pads. Prairie voles are born without fur, save fine whiskers protruding from whisker pads on their dorsolateral snouts, known as mystacial vibrissae. Somatosensory input from whiskers has been shown to modulate central OXT in developing mice, indicating a role for OXT signaling in somatosensory development and cortical plasticity (Zheng et al., 2014). Prairie voles administered intraperitoneal injections of OXT on the day of birth experienced a persistent reduction in the relative size of their S1 primary somatosensory cortex, an effect that was only observed in females (Seelke et al., 2016). However, we observed no significant OXTR net signal on the whisker pads of either male or female prairie voles.

Olfactory epithelial tissue surrounding nasal turbinates dorsal to the vomeronasal organ displayed faint but significant signal in male and female prairie voles (Figure 2). OXTR has been identified in several brain regions strongly associated with olfaction such as olfactory bulbs, olfactory tubercles, piriform cortex, entorhinal cortex, and the peduncular cortex (Gimpl and Fahrenholz, 2001). OXTR modulate olfactory bulb projections to several cortical regions and subcortical targets such as the piriform cortex, entorhinal cortex, bed nucleus of the stria terminalis, and the amygdala (Ross and Young, 2009; Stoop, 2012). However, data on OXTR in the peripheral tissues associated with chemosensory processing, such as olfactory epithelium and the vomeronasal organ, is less established. OXT has elicited excitatory effects on central neurons from brain regions receiving olfactory stimulation (Fang et al., 2008), and blocking OXTR activation has demonstrated disruption of chemosensory processing at cellular (Samuelsen and Meredith, 2011) as well as behavioral level (Martinez et al., 2010). A series of experiments in neonatal rats demonstrated effective olfactory conditioning within a 4-hour perinatal window (Miller and Spear, 2009). Human infants have similarly demonstrated preferential orienting toward odors associated with their mothers (Porter, 1999; Sullivan et al., 2011; Duran et al., 2013). Studies performed in rats demonstrated that centrally administered OXT can facilitate this type of odor imprinting (Kojima and Alberts, 2011), however, the effect of peripherally administered OXT on olfactory-driven development is unknown. Perhaps OXTR in the olfactory epithelium of neonates plays an important modulatory role in the rapid olfactory-based learning that drives social orienting behaviors in mammalian neonates.

The ciliary bodies of the eye displayed dense OXTR binding that competed away with unlabeled OXT (Figure 3). This region is responsible for the maintenance of the aqueous humor and contains the muscles controlling pupillary responses. The function of OXTR in this area is currently unknown, but there is evidence for OXT modulating autonomic processes across different mammalian species, with evidence for OXT to modulate both sympathetic and parasympathetic processes (Yee et al., 2016). Pupillary responses to environmental stimuli are known to be modulated by balanced control of both sympathetic and parasympathetic neural control (Neuhuber and Schrodl, 2011; McDougal and Gamlin, 2015). However, intranasal OXT administration has been shown to increase pupil dilation in humans, which is more specifically associated with sympathetic arousal (Leknes et al., 2013; Prehn et al., 2013; Ellingsen et al., 2014), but the mechanisms and pathways facilitating this effect are still unknown. *Oxt* and *Oxtr* mRNA has been previously identified in C57Bl6 mice, rhesus macaque, and human retinal tissue, suggesting an OXT signaling pathway (Halbach et al., 2015; York et al., 2017), although local generation of *Oxt* mRNA may be species-specific, as it was not present in the Sprague-Dawley rat retina (Tsuji et al., 2017). These binding patterns are similar to those observed in C57Bl/6J mice (Greenwood and Hammock, 2017), however, more specific cell type and functionality of OXTRs in this location are yet to be determined. While there is interesting preliminary evidence in the literature for a relationship between OXT/OXTR signaling in the eye as described, it is not clear what role it might play in a blind altricial neonate.

OXT receptors ligand binding in the adrenal gland was not significantly different from zero in males or females, with high variability in both sexes. This structure demonstrated intense signal that was not significantly competed off with unlabeled OXT. This contrasts with signal observed in the adrenal gland of the mouse, which was not present in the *Oxtr* knockout mouse and was significantly competed away in the wild-type by unlabeled OXT. Modulation of HPA axis responses by OXT have been noted across different species in multiple studies (Gibbs et al., 1984; Petersson et al., 1999; Heinrichs et al., 2003; Windle et al., 2004; Amico et al., 2008; Grippo et al., 2012; Smith and Wang, 2014), however, a lack of evidence correlating peripheral measures of OXT and adrenal hormones (Kenkel et al., 2012), suggests that OXT may be exerting these effects through primarily central regulation. However, many studies have examined nuanced effects of OXT on HPA axis response, noting differential outcomes based off species, sex, and nature of the stressor (Yee et al., 2016).

The anogenital area was measured at the skin rostral to the genitalia, stopping just anterior to the rectum on slices where internal and external genitals were clearly visible in the cresyl violet sections (Figure 4). Net values of OXTR ligand binding in this region were low but significantly different from zero in both male and female subjects with little variability. Anogenital licking has been observed across rodent species as an integral part of parental care (Gubernick and Alberts, 1987; Stern, 1996; Shoji and Kato, 2006). As measured in rats, this type of stimulation significantly influences the long-term neuroanatomical and behavioral outcomes of the offspring (Moore and Morelli, 1979; Moore, 1984; Caldji et al., 1998; Zhang et al., 2005). Prairie voles have been observed to engage in anogenital licking, however, it is unknown if this behavior correlates to the hippocampal and HPA axis modifications observed in rats (Suchecki et al., 1993; Liu et al., 1997; Francis and Meaney, 1999; Levine, 2002; Bredy et al., 2003; Weaver et al., 2006; Cameron et al., 2008). Maternal anogenital licking has also been associated with activation of oxytocinergic neural pathways in rats, rabbits, and mice, (Francis et al., 2000; Caba et al., 2003; Lenz and Sengelaub, 2010). Prairie voles are one of few rodent species to engage in biparental behaviors, which significantly influences species-typical development of the offspring (Ahern and Young, 2009; Ahern et al., 2011; Perkeybile et al., 2015). Like prairie vole mothers, fathers engage in anogenital licking of the offspring (Solomon, 1993; Lonstein and DeVries, 1999; McGuire et al., 2003), however, to date the effect of paternal licking and grooming on neuroendocrine development, specifically that of oxytocinergic systems, has not been studied. Similar to observations previously mentioned in other species, high levels of parental care are associated with high levels of *Oxtr* gene expression (Perkeybile et al., 2019).

OXT receptors net ligand binding was not significantly different from zero in males nor females in the liver. These data are congruent with previous data from our lab in *Oxtr* mice that demonstrated no significant specific ligand binding in the liver (Greenwood and Hammock, 2017), despite high raw film density values in that area. Similar to the mice, vole tissue demonstrated robust film density that was not competed away with unlabeled OXT, producing a net value not different from zero. The *Oxtr* knockout mouse exhibited markedly higher densitometry values than the wild-type counterpart suggesting that congenital loss of the OXTR gene changed the anatomy of the neonatal liver such that it demonstrated higher non-specific binding. Because we did not test an OXTR knock out prairie vole, it is not yet possible to say if congenital loss of OXTR in the prairie vole would alter prairie vole liver anatomy to increase the noise in the autoradiography. While it is clear that OXT does have significant interactions in metabolic processes, data within this paper do not support the presence of OXTR ligand binding in the liver in the neonatal prairie vole.

Brown adipose tissue (BAT) in the scapular region displayed significant OXTR ligand binding in female, but not male prairie voles (Figure 5). BAT is an important area for internal thermoregulation and may contribute to the development of mammals. Mice lacking OXT-signaling displayed difficulty maintaining core body temperatures in cold environments (Kasahara et al., 2007; Takayanagi et al., 2008). Rodents within a huddle exhibit preferences for warmer individuals, individuals that display greater BAT activation (Sokoloff and Blumberg, 2002), and in mice a preference for female pups was observed due to their higher BAT thermogenesis in comparison to males (Harshaw et al., 2014). *Oxt* knockout mice demonstrated impaired BAT thermogenesis in comparison to wild-type controls, and less preferential huddling toward knockout mice (Harshaw et al., 2018). Given the evidence that thermal regulation by huddling is an important behavior for young rodents, this raises the question as to whether BAT and its implications for thermogenesis have direct effects on social or neurobiological development. Previous work from our lab failed to detect specific OXTR ligand binding in the BAT of the neonatal mouse, despite high levels of signal on film. This signal on film was not competed off with unlabeled OXT and had higher densitometry values in the knockout compared to the wild-type mouse. In contrast, data in this paper provide evidence for the expression of OXTR in BAT in neonatal voles, specifically in females.

## Limitations and Future Directions

These results have technical and study design limitations that require further investigation. It is important to acknowledge that data presented within this paper represents a single time point, the date of birth. Replication with multiple age points would need to be performed to assess if these sites of peripheral OXTR undergo developmental changes, and/or persist into adulthood. Additionally, a larger sample size for males and females could potential identify more subtle sex differences. A larger sample size would also permit a more thorough analysis of additional regions. Laboratory prairie voles are recent descendants from wild caught voles and are maintained through outbreeding. Therefore, the potential for individual variability is higher than in inbred mouse strains. Intraspecies behavioral differences have been observed in prairie voles from distinct regional stocks (Roberts, 1998). A larger sample size, and in particular, a comparison of different stocks may reveal the potential for individual differences in peripheral expression of OXTR that may play a role in individual differences in developmental trajectories.

Receptor autoradiography methods and the radioligand used have high specificity for OXTR, however, non-specific signal occurred with high variability in peripheral tissues with high lipid content in a manner dissimilar from that of ligand binding previously observed in neural tissue (Hammock and Levitt, 2013). Radioligand affinity for tissue with high lipid content may be masking subtle differences in the binding patterns of OXTR. While this technique can give regionally specific precision, it does not provide cellular level resolution. Additional analyses are needed, especially in regions of interest such as the eyes, the oronasal cavity, or the anogenital area; to determine if the receptors are expressed by peripheral neurons, epithelium, muscle fibers, or other types of cells.

The influence of OXT on early social development has been demonstrated in animal models (Teng et al., 2013; Simpson et al., 2014), as well as human children (Yatawara et al., 2016; Tauber et al., 2017), suggesting a role for early organization of the developing social brain (Hammock, 2014; Kenkel et al., 2014). Thus far, research across different species has indicated a positive relationship between infant OXT, both centrally and peripherally, and maternal care (Noonan et al., 1994; Francis et al., 2000; Winslow et al., 2003; Kojima and Alberts, 2011; Mogi et al., 2011; Kojima et al., 2012). These same models also demonstrate the impact of maternal care on adaptive long-term behavioral outcomes for the infant (Liu et al., 1997; Caldji et al., 1998; Francis and Meaney, 1999; Bredy et al., 2003; Zhang et al., 2005; Weaver et al., 2006). However, the mechanisms by which maternal care exerts effects on infant OXT are unknown. Peripheral OXTR levels in the infant may be an important species-specific regulator of the transmission of parental care to the offspring. While OXT and the OXTR facilitate a mother’s ability to deliver, nourish, and nurture her offspring, OXT is found in maternal fluids that come into contact with offspring, such as amniotic fluid (Dawood et al., 1979), saliva (White-Traut et al., 2009; Feldman et al., 2010), and breast milk (Takeda et al., 1986). The infant may be able to detect maternal (or paternal) sources of OXT through peripheral OXTR.

## Data Availability

All datasets generated for this study are included in the manuscript and/or the Supplementary Files.

## Ethics Statement

All procedures were performed under protocols approved by the Institutional Animal Care and Use Committee of Florida State University in accordance with state and federal guidelines (Guide for the Care and Use of Laboratory Animals of the National Institutes of Health).

## Author Contributions

Both authors conceived and designed the experiment and wrote the manuscript. MG performed the experiment and analyzed the data.

## Conflict of Interest Statement

The authors declare that the research was conducted in the absence of any commercial or financial relationships that could be construed as a potential conflict of interest.

## Supplementary Material

Table S1 OXTR ligand binding density measurements in tissue sections after exposure to 0.05 nM radioactive ligand in male and female neonatal prairie voles competed with 0 nM or 1000 nM OXT. Net values represent the difference between 0 nM and 1000 nM competition and are used to demonstrate ligand specificity.
